# Determinants of Utilization of Health Extension Workers in the Context of Scale-Up of Integrated Community Case Management of Childhood Illnesses in Ethiopia

**DOI:** 10.4269/ajtmh.14-0660

**Published:** 2015-09-02

**Authors:** Bryan Shaw, Agbessi Amouzou, Nathan P. Miller, Amy O. Tsui, Jennifer Bryce, Mengistu Tafesse, Pamela J. Surkan

**Affiliations:** Department of International Health, Institute for International Programs, Johns Hopkins Bloomberg School of Public Health, Baltimore, Maryland; Department of Population Family and Reproductive Health, Johns Hopkins Bloomberg School of Public Health, Baltimore, Maryland; ABH Services PLC, Addis Ababa, Ethiopia

## Abstract

Ethiopia has invested significant resources in integrated community case management (iCCM) of childhood illness. In Oromia Region, iCCM scale-up was phased in, allowing for comparisons between districts providing iCCM and routine services. We assessed the determinants of utilization of health extension workers (HEWs) delivering iCCM services at rural health posts by caregivers of sick, under-five children in a cross-sectional survey. We found low utilization of HEWs with only 9.3% of caregivers of a child sick with diarrhea, fever, and/or pneumonia in the previous 2 weeks taking their child to HEWs in both iCCM and routine areas. There was a higher likelihood of utilization of HEWs in iCCM areas (OR: 1.44; 95% CI: 0.97–2.12; *P* = 0.07), but this effect disappeared after accounting for confounders. In iCCM areas, maternal education, illness type, and distance were associated with utilization. Perceptions of illness severity and service quality were the primary reasons given for not utilizing the health post. Our findings suggest that though iCCM is reaching some vulnerable populations, there remain significant barriers to use of HEWs delivering iCCM services. Efforts for demand generation and minimization of remaining barriers are urgently needed for the sustained success of the iCCM strategy in Ethiopia.

## Introduction

Diarrhea, malaria, and pneumonia are three of the leading causes of mortality in children under-5 years of age in sub-Saharan Africa.^1^,^2^ Empirical evidence demonstrates that the use of evidence-based treatments targeting these illnesses could prevent a large portion of child deaths.^3^,^4^ In Ethiopia, considerable progress has been made in improving access to essential public health interventions and in reducing under-five deaths in the past 10 years.^5^,^6^ Nevertheless, nearly 200,000 under-five children continue to die in Ethiopia every year, many due to preventable causes. These deaths are often concentrated in vulnerable and underserved populations.^2^

Following a policy change supporting community case management (CCM) of pneumonia in addition to existing CCM programs for diarrhea and malaria in 2010, Ethiopia has been scaling-up integrated CCM (iCCM) of common childhood illnesses in six regions within the country within the national Health Extension Program (HEP). Central to the iCCM strategy in Ethiopia, health extension workers (HEWs) are trained to use an algorithm to treat common child illnesses, provided with a regular supply of oral rehydration salts (ORS), antimalarial drugs, and antibiotics, and are deployed in community health posts. In particular, the iCCM strategy aims to improve access to these treatments by providing free treatments to the largely rural populace, often lacking effective access to private care, public health centers, and higher-level facilities.^7^,^8^ This strategy's goals are to improve equity and under-five mortality outcomes.^9^

Despite increasing access, utilization of public services providing evidence-based services remains frustratingly low in many regions of Ethiopia.^10^,^11^ Use of HEWs for treatment of child illnesses has been found to be very low. Estimates of utilization of HEWs for sick children range from 4% to 17% for children sick in the previous 2 weeks, with large variation between regions and by illness.^12^,^13^ Multiple factors related to affordability, geographic accessibility, and availability have been identified as influential in caregiver decisions to use HEWs for treating child illness.^14^

At such low levels of utilization it is unlikely that Ethiopia's efforts are achieving their full potential in reducing under-five deaths. Since the implementation of the HEP and iCCM strategies, few published studies have explored the reasons for low levels of utilization and the effectiveness of these programs in promoting the use of evidence-based child health services.^10^,^11^,^15^ Furthermore, very few studies have looked at the effects of these programs by specific subpopulations,^15^,^16^ and it is uncertain whether they are reaching the most vulnerable subpopulations, promoting equity as intended.

In the context of low utilization despite increasing availability of free services provided by HEWs within rural Ethiopian communities, it is essential to explore social and economic determinants and patterns in health services utilization.^15^,^16^ The Andersen Health Services Utilization Framework suggests these determinants can be organized into three dynamics: 1) predisposing characteristics existing before an illness (e.g., age and health beliefs); 2) enabling characteristics relating to the logistical aspects of obtaining care (e.g., geographic distance and socioeconomic status [SES]); and 3) need characteristics relating to judgments about illness and care (e.g., perceptions about illness severity).^17^,^18^ An understanding of these determinants is a prerequisite to strengthening health systems and attaining universal and equitable child health services through the identification and amelioration of remaining access barriers and vulnerable populations. This study seeks to fill the gap in knowledge of patterns of care seeking from HEWs delivering evidence-based treatments for sick children in community-based settings and their social and economic determinants in the context of scale-up of iCCM services.

## Materials and Methods

### Study area.

The study was conducted in Jimma and West Hararghe zones (districts) of Oromia Region in central Ethiopia. These zones were chosen because of their relatively large populations, strong presence of iCCM implementation partners, and phased implementation of the iCCM program. Jimma and West Hararghe zones have populations of approximately 2.9 and 2.1 million people, respectively.^19^ In the two zones, the population is predominantly rural, Muslim, and of Oromo ethnicity. The primary occupation is subsistence agriculture.^20^ The terrain is largely mountainous and mostly forested in Jimma and semiarid in West Hararghe. The major rainy season extends from June to September. Malaria risk varies in the study areas, with about one-quarter of health posts in high-malaria risk areas.

### Survey design and inclusion criteria.

We conducted a cross-sectional survey of households in the two study zones. As part of the phase-in process, rural *woredas* (subdistricts) were randomly assigned to receive either the iCCM intervention or the preexisting, routine CCM comparison program. Table 1 provides a comparison between routine CCM and iCCM guidelines and inputs. Woredas providing the iCCM program were fully implementing iCCM as of July 2011, approximately 2 years before the conduct of the survey.

The survey used a stratified, two-stage cluster sampling design with clusters represented by the 2007 census enumeration areas (EAs) and strata represented by rural woredas. Information for EAs was obtained from the Central Statistical Agency of Ethiopia. In the first stage of sampling, EAs within each woreda were selected using systematic random sampling with probability proportionate to size. Households in each selected EA were listed, and a subset of 35–36 households was randomly selected for inclusion using systematic random sampling. Heads of households were interviewed to obtain a list of all members of the household. Eligible women aged 15–49 years and self-identified primary caregivers of under-five children were selected for interviews.

### Sample size and study sample.

The sample size for the survey was determined using the indicator of treatment of acute respiratory infection (ARI) among children under-five. Based on this indicator, 6,000 households were needed each for the intervention and comparison areas to measure a level of treatment of ARI among under-five children of 50% with an absolute precision of 6%. This gave a total sample of 12,000 households.

The sample for this study consists of all under-five children residing in or having slept in a selected household on the previous night and identified as having been sick with diarrhea, fever, and/or pneumonia in the 2 weeks before the interview. The child's primary caregiver completed the interview. A single caregiver could be interviewed for multiple children that met this condition. Children with diarrhea or fever were identified based on the reported presence of those symptoms. Presence of pneumonia was presumed and based on responses to a series of questions identifying the presence of cough and faster than usual breathing with difficulty breathing due to chest problems.

### Survey instruments.

The survey was composed of three modules: a household, woman of reproductive age (WRA), and under-five questionnaire. The head of household was interviewed for the household questionnaire to list all household members and demographic information. The WRA questionnaire, for women aged 15–49 years, included the woman's demographic information and birth history. The under-five survey was completed by a child's primary caregiver and included sub-modules on child demographic information and for the prevalence and practices associated with cough/fever and diarrhea.

The modules and interview questionnaires were based primarily on the Demographic and Health Survey and the Multiple Indicator Cluster Survey. These modules were modified to include specific domains of interest. The main modification was the addition of a care-seeking module in the under-five questionnaire. Questionnaires were developed in English and translated into Afan Oromo and Amharic. Questionnaires were pretested and piloted in rural Oromia woredas located near Addis Ababa. Several translation iterations were conducted based on feedback from the pretest and over the course of training to refine the instruments in terms of clarity of concepts and translation.

### Data collection.

A total of 120 data collectors who had completed high school, had experience in doing survey interviews, and were fluent in English and Afan Oromo were trained for 3 weeks. Twenty of the highest performing data collectors were selected as supervisors to head 20 teams of one supervisor and four data collectors each. Permission to conduct interviews was obtained by zonal and woreda health bureaus before data collection for each site. Each team spent approximately 1 day mapping an EA and 1–2 days conducting interviews. Data were collected on laptop computers using a computer-assisted personal interviewing feature of Census and Survey Processing System (CSPro) 5.0 software (U.S. Census Bureau, Washington, DC). Three staff members from the Institute for International Programs at Johns Hopkins University (IIP/JHU), including the first author (Bryan Shaw), were present to provide additional support and monitoring. Team supervisors and IIP/JHU staff conducted random quality control checks. Data was incorporated directly into a central electronic database in Addis Ababa. Validity and consistency checks were run in CSPro to check entered data. Data collection took place for approximately 5 months from February to July 2013.

### Measures.

The primary outcome of interest for this study was the reported utilization of the HEW at the health post by a caregiver of an under-five child sick with diarrhea, fever, and/or suspected pneumonia in the 2 weeks preceding an interview (*N* = 2,248). We focused specifically on utilization of HEWs at the health post rather than including higher levels of care (e.g., health centers) as HEWs are the key entry point for caregivers of sick children in rural areas with low access to higher levels of care and central actors in the success of the iCCM strategy in Ethiopia.^9^ A key independent variable of interest was residence of the caregiver and child in woredas providing either routine CCM services or iCCM services.

Covariates considered in this analysis were grouped into 1) predisposing characteristics: religion, household size, marriage status, maternal age, maternal education, maternal literacy, previous use of health post services, and child gender; 2) enabling characteristics: zone of residence, household wealth and awareness of the availability of treatments for child illness at the health post; and (3) need characteristics: maternal knowledge of child illness danger signs, previous experience of a child death, child age, and illness type. In the Andersen framework, categorizations were based on hypothesized relationships between independent variables and the use of the HEW at the health post for a sick child.^17^,^18^,^21^ Household wealth was grouped into wealth quintiles based on index scores constructed for each household using principal component analysis of household assets, income sources, and housing characteristics.^22^ Considering the similarities between quintiles in rural, low-income settings as suggested by Agho and others,^23^ the wealth quintiles were recategorized into three groups: 1) lowest 40%, 2) middle 40%, and 3) upper 20%. Distance from the nearest health facility or health post was based on reported travel time, which has been suggested as a superior measure to direct distance.^24^,^25^

### Data analysis and model selection.

Data were entered into CSPro, and consistency and validation checks were conducted. Data analysis was performed using STATA version 13 (StataCorp, College Station, TX). Exploratory data analyses were conducted to examine the extent of missing data and dispersion of the outcome and explanatory variables. Factors associated with utilization of the HEW at the health post were examined using bivariate and multivariate logistic regression. All descriptive statistics and logistic regressions were computed to account for the complex design and nested structure of the data through weighting of observations by the inverse probability of selection and using the Taylor linearization procedure for computing standard errors.^26^ Strong collinearity was found between caregiver literacy status and education, and literacy status was dropped from the analysis. Caregiver marriage status was also dropped because of very low numbers of unmarried caregivers.

For multiple logistic regression, independent variables were introduced in blocks with predisposing characteristics entered first (model 1) followed by predisposing + enabling characteristics (model 2) followed by predisposing + enabling + need characteristics (model 3). Previous use, awareness of the availability of treatments for child illness, and maternal knowledge of danger signs were not included in multiple logistic regression models as they were likely to be influenced by the type of child health intervention (routine CCM versus iCCM) and considered endogenous variables. To evaluate the effect of the iCCM intervention on utilization of the HEW at the health post, adjusted odds ratios (aORs) were evaluated for this independent variable of interest in each of the models accounting for potential confounders. To evaluate significant factors associated with utilizing the HEW at the health post unique to routine CCM versus iCCM woredas, logistic regression models using the block procedure were also run after stratifying the sample according to type of child health services available. The relationship between predictor variables and the outcomes of interest were considered marginally significant at the *P* < 0.10 level and significant at the *P* < 0.05 level.

Multinomial logistic regression analyses were conducted to examine factors associated with utilization of one type of care source among multiple options. The outcome of interest for these analyses was choice of health-care source grouped into 1) no care/home (this includes informal care such as herbal remedies and treatments obtained through local vendors) care, 2) HEW at the health post, 3) health center, and 4) private care source. Three multinomial regression models were run using the following reference groups: 1) no care/home care, 2) HEW at the health post, and 3) private care source. This strategy generated six models of all possible comparisons between sources. In particular, multinomial logistic regression was performed to assess differences in patterns of use of these sources comparing iCCM woredas to routine CCM woredas. Independent variables that were hypothesized to impact choice among these alternatives and those that were largely exogenous to the decision were included in multinomial logistic regression models. These variables included type of child services available (routine CCM versus iCCM), maternal education status, child gender, zone of residence, household wealth, household distance from the health post, and previous experience of a child death. Multinomial logistic regression results were given in adjusted relative risk ratios (aRRRs).

### Ethical considerations.

Ethical approval was obtained from the institutional review boards (IRBs) of the Oromia Regional Health Bureau and the Johns Hopkins University Bloomberg School of Public Health. Informed oral consent was obtained from all participants. Written consent from all participants would not have been possible because many of the participants were not literate. The IRBs approved the use of oral consent, and data collectors noted on a form whether oral consent was obtained from each participant.

## Results

### Demographic characteristics of study participants.

Participants indicated a total of 2,248 caregivers of children sick with diarrhea, fever, and/or suspected pneumonia in the 2 weeks before the interview. Selected predisposing, enabling, and need characteristics collected for the sample are presented in Table 2. Of particular note, less than half (43.1%) of all caregivers interviewed were aware of the availability of treatments for child illness at the health post and nearly three-quarters (70.2%) of caregivers had never visited the health post. Of the sick children, 44.5% of caregivers reported the presence of diarrhea, 69.2% fever, and 24.4% pneumonia. Nearly one-third (32.3%) reported more than one of these illnesses for their child.

A total of 1,247 children were reported sick with diarrhea, fever, and/or suspected pneumonia in the previous 2 weeks in woredas providing routine CCM services compared with 1,001 children in woredas proving iCCM services. There were a few statistically significant differences in characteristics between these settings. Significant differences were detected for knowledge of danger signs and awareness of the availability of child health services at the health post with higher proportions of caregivers in iCCM woredas reporting awareness of the availability of child health services (48.6% versus 37.3%) at the health post and mentioning a larger number of child illness danger signs (3.4 versus 2.5) compared with caregivers in routine CCM woredas. There were no differences observed in previous use of the HEW by caregivers in iCCM compared with routine CCM areas.

### Care-seeking sources.

**Figure 1.**
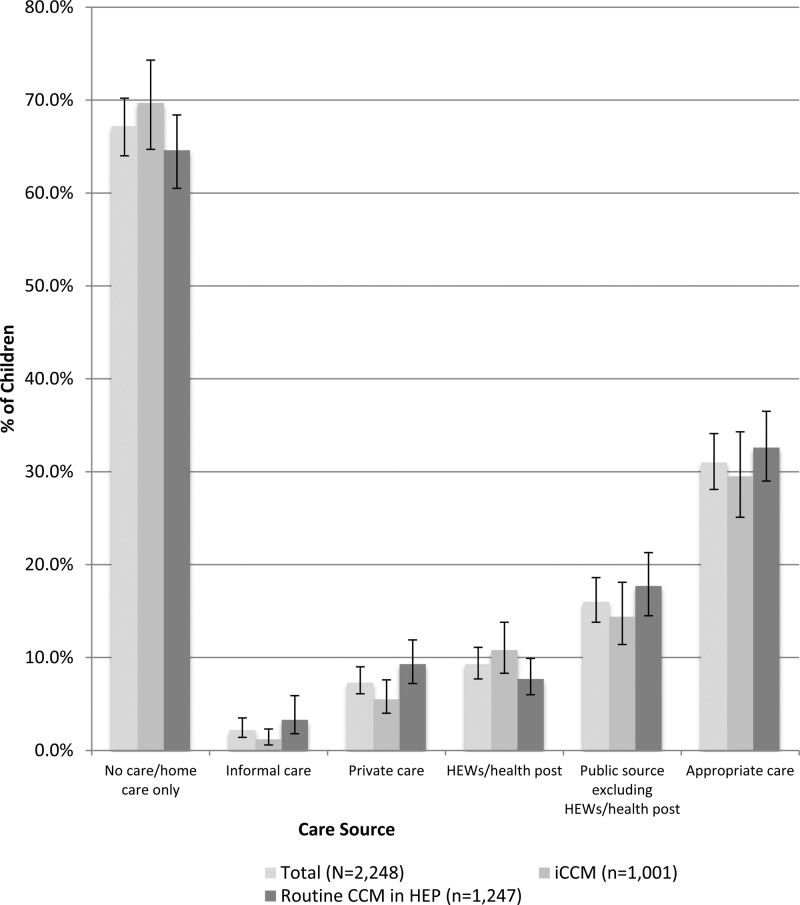
Care-seeking sources used for children reported sick with diarrhea, fever, and/or pneumonia in the 2 weeks preceding the survey in Jimma and West Hararghe zones, Oromia Region, Ethiopia, 2013 (*N* = 2,248).

Reported care-seeking sources by caregivers of a child sick with diarrhea, fever, and/or pneumonia in the 2 weeks preceding the survey are given in Figure 1

. Of the 2,248 caregivers in this analysis, 643 (31.0%) reported seeking care from an appropriate source—a source where evidence-based child health treatments could, in theory, be obtained. Among appropriate sources, 188 (9.3%) sought care from HEWs at the health post, 335 (16.0%) from a higher-level public source, and 154 (7.3%) from a private source.

Differences in appropriate care seeking between caregivers residing in woredas providing iCCM services (29.5%) and caregivers residing in woredas providing routine CCM services (32.6%) were not statistically significant. Differences in utilization of the HEW at the health post were marginally significant (*P* = 0.07) between caregivers residing in woredas providing iCCM services (10.8%) and caregivers residing in woredas providing routine CCM services (7.7%).

### Utilization of HEWs at the health post.

Of the 2,248 caregivers in this analysis, 188 (9.3%) sought care from the HEW/health post over the course of their child's illness. By intervention type, 95 (7.7%) caregivers used the HEW at the health post in routine CCM woredas compared with 93 (10.8%) in iCCM woredas. Table 3 presents the results of logistic regression models for care seeking from the HEW at the health post comparing caregivers in routine CCM woredas to those in iCCM woredas. In the univariate logistic regression model, caregivers in iCCM woredas were more likely (OR: 1.44; 95% CI: 0.97–2.12; *P* = 0.07) to use the HEW at the health post compared with caregivers in routine CCM woredas. No significant relationships were observed after accounting for predisposing (aOR: 1.35; 95% CI: 0.91–2.00; *P* = 0.13), enabling (aOR: 1.35; 95% CI: 0.91–1.99; *P* = 0.14), and need characteristics (aOR: 1.33; 95% CI: 0.89–1.97; *P* = 0.16) in models 1, 2, and 3, respectively.

### Determinants of utilization of HEWs at the health post.

In bivariate analyses (Table 4), previous use of the health post for child illness (OR: 6.01; 95% CI: 3.84–9.40; *P* < 0.01) and awareness of the availability of treatments for child illness at the health post (OR: 2.52; 95% CI: 1.64–3.84; *P* < 0.01) were the strongest predictors of health post use for caregivers in both routine CCM and iCCM woredas. Increasing distance of a household from the health post was significantly associated with a lower likelihood of using the health post with caregivers residing a 30- to 60-minute walk (OR: 0.66; 95% CI: 0.42–1.04; *P* < 0.10), a 1- to 2-hour walk (OR: 0.61; 95% CI: 0.39–0.95; *P* < 0.05), and greater than 2-hour walk (OR: 0.55; 95% CI: 0.30–0.99; *P* < 0.05) all significantly less likely to use the health post compared with caregivers residing less than a 30-minute walk to the health post. Caregivers in West Hararghe were also less likely to use the health post (OR: 0.70; 95% CI: 0.47–1.04; *P* < 0.10) compared with caregivers in Jimma zone.

Table 5 compares determinants of utilization of the HEW at the health post between routine CCM and iCCM woredas after accounting for confounders. In the final model accounting for predisposing, enabling, and need factors in routine CCM woredas, significant relationships were found only for maternal age and household distance from the health post with the use of the HEW at the health post for a sick child. In routine CCM areas, younger caregivers (age 15–19 years) were less likely (aOR: 0.06; 95% CI: 0.01–0.58; *P* < 0.05) to use the health post compared with caregivers of age 20–29 years. Children living in households within 31–60 minutes travel time were less likely (aOR: 0.51; 95% CI: 0.24–1.07; *P* < 0.10) to be taken to the health post compared with children living within 30 minutes travel time to the health post.

In iCCM woredas in the final model, maternal education, child age, and illness type were all significantly related to the use of the health post. In contrast to determinants in CCM woredas, no significant relationships were found in the use of the health post and categories of maternal age. However, mothers with some formal education had lower odds (aOR: 0.46; 95% CI: 0.26–0.79; *P* < 0.01) for using the health post compared with mothers with no formal education. For distance, in contrast to caregivers in CCM woredas, there was a steady decreasing odds for using the health post as distance increased with caregivers residing between 1 and 2 hours from the health post were less likely (aOR: 0.31; 95% CI: 0.15–0.69; *P* < 0.01) and caregivers residing more than 2 hours were less likely (aOR: 0.29; 95% CI: 0.11–0.73; *P* < 0.01) to use the health post compared with caregivers residing less than a 30-minute walk from the health post. Significant relationships were also found for child age and illness type for the use of health post services. In contrast to CCM woredas, children in iCCM woredas who were under the age of 1 year were significantly less likely (aOR: 0.31; 95% CI: 0.13–0.74; *P* < 0.01) to be taken to the health post. Finally, children with reported pneumonia were more likely (aOR: 2.69; 95% CI: 1.30–5.56; *P* < 0.05) to be taken to the health post compared with children with reported diarrhea only. No significant relationships were seen by household wealth.

### Determinants of utilization of sources in multinomial regression models.

Table 6 reports results from the multinomial regression models examining the association between type of child health services and selected sociodemographic and socioeconomic factors and type of source used for a sick child. Relative to caregivers in CCM woredas, those in iCCM ones were less likely to use the health center (aRRR: 0.65; 95% CI: 0.39–1.09; *P* < 0.10) and private sources of care (aRRR: 0.44; 95% CI: 0.25–0.76; *P* < 0.01) compared with using the health post, suggesting that the iCCM strategy was leading to use of health posts over higher levels of care.

Looking at sociodemographic factors in multinomial regression models, maternal education was significantly associated with increased likelihood of using the health center (aRRR: 1.69; 95% CI: 1.20–2.38; *P* < 0.01) and private sources (aRRR: 2.41; 95% CI: 1.56–3.73; *P* < 0.01) compared with home care only, while there was no difference between the use of the health post compared with the use of home care only by maternal education. Maternal education was also significantly associated with increased likelihood of using the health center (aRRR: 2.01; 95% CI: 1.13–3.59; *P* < 0.05) and private sources of care (aRRR: 2.86; 95% CI: 1.60–5.12; *P* < 0.01) compared with the health post after controlling for selected factors and type of child health intervention. There were differences observed between zones with caregiver in West Hararghe less likely (aRRR: 0.66; 95% CI: 1.42–1.05; *P* < 0.10) to use the health post relative to home care only. In addition, caregivers in West Hararghe were more likely (aRRR: 1.84; 95% CI: 0.95–3.57; *P* < 0.10) to use private sources relative to the health post. Household distance from the health post was generally associated with lower likelihood of using both the health post and the health center relative to home care only. For example, caregivers residing between a 1- to 2-hour walk were approximately half as likely to use both the health post (aRRR: 0.52; 95% CI: 0.33–0.84; *P* < 0.01) and health center (aRRR: 0.52; 95% CI: 0.36–0.76; *P* < 0.01) compared with caregivers residing less than a 30-minute walk relative to home care only.

### Reported reasons for not seeking care from the HEW/health post.

**Figure 2.**
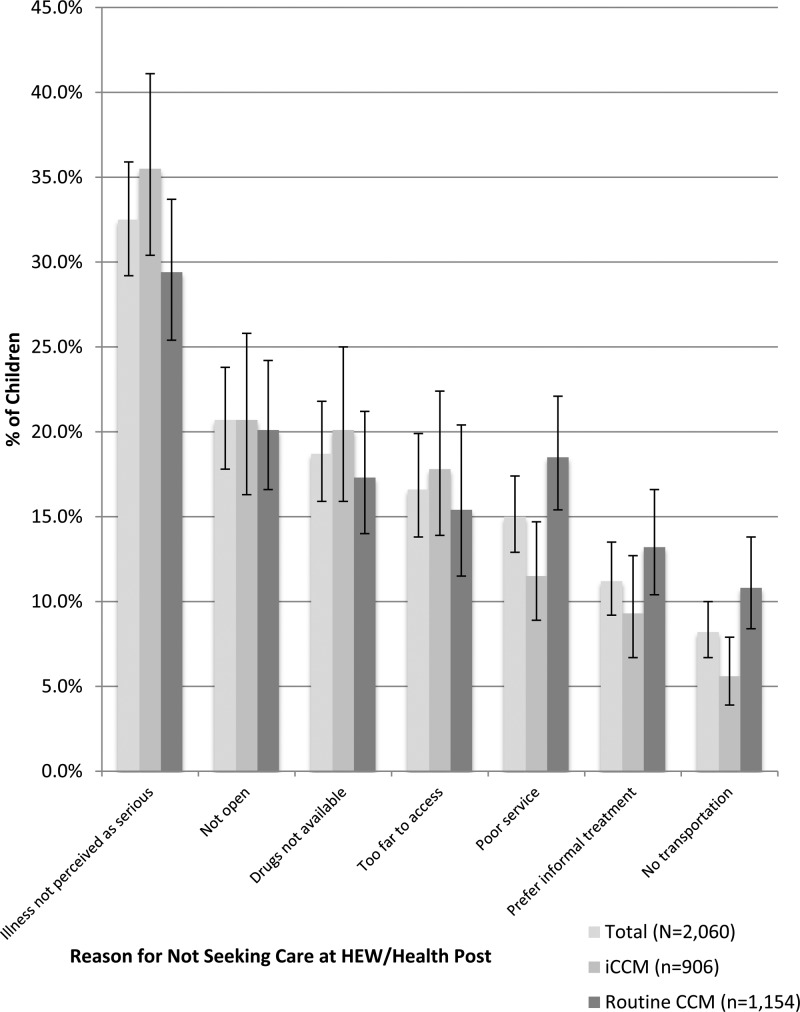
Reported reasons for not seeking care from the health extension worker (HEW)/health post among caregivers not utilizing the health post for children reported sick with diarrhea, fever, and/or pneumonia in the 2 weeks preceding the survey in Jimma and West Hararghe zones, Oromia Region, Ethiopia, 2013 (*N* = 2,060).

The primary reasons reported by caregivers who did not use services from the HEW at the health post (*N* = 2,060) for not using this source for their child's illness are presented in Figure 2

. The most common response to this question related to perceptions that the child's illness was not serious enough to go to the health post. Nearly one-third of these caregivers (32.5%) reported this perception. Additional reasons included perceptions that the health post was not open (20.7%), drugs were not available at the health post (18.7%), the health post was located too far from a caregiver's household to access (16.6%), poor service by the HEW or at the health post (15.0%), preferences for informal treatment (11.2%), and challenges in obtaining transportation to the health post (8.2%).

Comparing caregivers in woredas providing iCCM services to caregivers in those providing routine CCM services, highly significant differences (*P* < 0.01) were found for caregivers reporting poor service by the HEW or at the health post and challenges in obtaining transportation to the health post. Higher proportions of caregivers in woredas providing routine CCM services reported poor service (18.5%) and transportation challenges (10.8%) as reasons for not using the health post compared with caregivers in woredas providing iCCM services (11.5% and 5.6%, respectively). Marginally significant differences (*P* < 0.10) were found for caregivers reporting perceptions that their child's illness was not serious, with higher proportions reporting this reason for not using the health post in woredas providing iCCM services (35.5%) compared with those in woredas providing routine CCM services (29.4%). Marginally significant differences (*P* < 0.10) were also found in caregivers stating preferences for informal treatment as a reason for not using the health post with higher proportions of caregivers in woredas providing routine CCM services (13.2%) reporting this reason compared with caregivers in woredas providing iCCM services (9.3%).

## Discussion

Several countries and international agencies are advocating for rapid scale-up of iCCM delivered largely through community health workers (CHWs).^27^ The scale-up of iCCM in Ethiopia is among the most ambitious implementations of iCCM in sub-Saharan Africa to increase coverage of evidence-based child health treatments, improve equity, and, ultimately, decrease under-five mortality. The country is making significant gains in reducing under-five mortality and has several advantages including a large cadre of literate, trained, and salaried HEWs.^28^ However, it is unlikely that the investments in iCCM scale-up are achieving their full potential given such low use of HEWs, a central component to delivery of iCCM. Furthermore, it is uncertain that iCCM is reaching caregivers and children most in need of services. For these reasons, it is important to take stock of utilization patterns in an effort to improve the impact of the iCCM strategy in Ethiopia as well as other low-income nations.

Utilization of health services relates to both supply- and demand-side factors.^18^,^21^ In a recent study by Miller and others^29^ assessing quality of care and implementation strength associated with the iCCM scale-up in Oromia, the authors found generally high capacity of HEWs to correctly manage child pneumonia, diarrhea and malnutrition, high rates of HEW supervision and training, and regular availability of essential child health commodities at the health post. Despite generally high performance in supply-side indicators, the authors in this study did find indications of supply-side barriers such as health posts being closed on weekends and open for an average of only 20 hours per week and HEW time devoted to active case finding.^29^ In our study conducted in the same geographic setting, we found that a major reason given by caregivers for not using the health post was a perception of lack of drugs suggesting a potential disconnect or lag between the community perceptions and health post capacity. We also found perceptions about the health post or HEW not being available as a major reason given for not using the health post for a sick child. Taken together, the quality of care study in Oromia^29^ and our study suggests the need for efforts aimed at understanding and promoting community demand as well as continual, targeted improvements in reducing supply-side barriers to access the iCCM strategy.^29^

HEWs delivering iCCM services at the health post are only one care option for a caregiver with a sick child in rural Ethiopia. For rural caregivers, public health centers and private drug vendors, pharmacies, and clinics are also used for treating sick children.^7^,^10^ However, these facilities often cluster in urban or semi-urban locations and are effectively out of reach for a large portion of the population distant from these urban settings.^7^ The iCCM strategy is explicitly designed to reduce these barriers, promoting equity in addition to reductions in child mortality.^9^ Nevertheless, despite significant investments in this service delivery strategy, little is known about patterns of utilization of CHWs by these populations and influential factors such as demand and access barriers to utilization in these settings.^30^,^31^

We found very low rates of utilization (9.3%) of HEWs for the total sample of caregivers in both iCCM and CCM areas of children sick with diarrhea, fever, and/or pneumonia after 2 years of scale-up of the iCCM strategy in rural Oromia Region. This low utilization stands in stark contrast to recent studies in sub-Saharan African demonstrating a range of utilization rates of CHWs for child illness from 26% to 78%.^32^–^37^ This low utilization occurs despite the provision of free services provided in local, community settings and status of HEWs as paid, government employees—intervention actions designed to minimize common barriers affecting CHW programs and their utilization.^9^,^28^,^38^

There were a slightly higher proportion of caregivers in iCCM woredas (10.8%) using HEWs compared with caregivers in routine CCM woredas (7.7%). However, after accounting for a range of potential confounders, no significant differences were seen in likelihood of use of HEWs in iCCM areas (aOR: 1.33; 95% CI: 0.89–1.97; *P* = 0.16) compared with routine CCM areas. In contrast, limited scale-ups of iCCM were found to significantly improve utilization of CHWs in studies in Kenya,^32^ Uganda,^33^–^35^ and Zambia^39^ relative to CCM of only one illness of a child.

There were some significant differences in the study population between caregivers in routine CCM woredas compared with caregivers in iCCM woredas. Notably, caregivers in iCCM woredas were more likely to be aware of treatments for child illness at the health post and had a greater knowledge of child illness danger signs. Although it is likely that the iCCM intervention is leading to increased caregiver knowledge of child illness and awareness of HEW capacities through the added emphasis on child illnesses, it does not appear that these are translating to increased use relative to the routine CCM program as has been observed for other studies on health services utilization in Ethiopia.^8^,^40^,^41^ A recent quality of care study conducted in Oromia showed significant improvements in several supply-side indicators in iCCM woredas relative to routine CCM woredas.^29^ Similarly, it does not appear that quality improvements in service delivery in iCCM areas have led to increased utilization of HEWs providing iCCM services in rural Oromia health posts after 2 years of scale-up.

There was a differing profile of determinants of utilization of HEWs at the health post comparing iCCM to routine CCM woredas. In both areas, previous use and awareness of the availability of treatments at the health post were associated with significantly higher odds for using the health post in unadjusted analyses, but the effect for both was stronger in iCCM woredas. The main differences found consisted of higher odds for the use of HEWs in iCCM woredas for younger mothers, mothers with no education, and children with pneumonia compared to routine CCM woredas. This suggests that the iCCM strategy is potentially reaching some groups typically facing barriers to accessing evidence-based child health treatments and that the inclusion of pneumonia in iCCM is leading to more community-based treatment of this illness.

In our study, caregivers living greater than a 1-hour walk from the health post were less likely to use HEWs in iCCM areas compared with CCM areas. This effect of distance on utilization of iCCM services has also been observed in Uganda where CHWs are expected to be the first line of contact within the health system.^34^ The authors suggested that it is possible that the added responsibilities of CHWs in the iCCM strategy could be interfering with their ability to extend services to households distant from the health post, particularly in their demand generation and community mobilization activities. This mechanism could also be responsible for this observed trend in Ethiopia as HEW responsibilities have been significantly increased by the iCCM strategy.^9^,^11^,^28^

We found no significant associations between household wealth and utilization of HEWs for child illness in either iCCM or CCM areas. Our finding contrasts with studies in Kenya^32^ and Uganda,^33^ which showed increased use of iCCM-trained CHWs by poorer households relative to wealthier households. This may be related to the relatively small differences in assets between the wealthiest and poorest households and the lack of a significant relationship in iCCM areas to the relatively short follow-up period from full implementation of iCCM. In some studies in rural Ethiopia, educational status has been suggested as a better proxy for SES compared with household income.^24^,^30^ Taking maternal education as a proxy for SES in our study, it appears lower socioeconomic households are more likely to use HEWs in iCCM areas compared with such households in routine CCM areas. This finding suggests promising effects on iCCM's promotion of equity in access to community-based child health services.

The strategy of iCCM is expected to improve policy support, quality, access, and demand for child health services and treatments for common childhood illnesses from trained CHWs, improve equity in access to evidence-based treatments, and reduce under-five mortality.^7^ There are some indications that the iCCM strategy is promoting increased access for marginalized populations such as uneducated caregivers and promoting equity. However, the low utilization seen in this study suggests that there are still access challenges such as distance of a household from a health post and that overall level of demand is generally low. In addition, the findings relevant to education and outreach activities aimed at awareness of child illness are mixed. Although caregivers in iCCM areas were more aware of the availability of treatments and child illness danger signs, nearly one-third of caregivers who did not use the health post in both areas stated that they did not use the health post because of low perceptions of severity of their child's illness—despite being reported sick with diarrhea, fever, and/or pneumonia. Furthermore, many caregivers who did not use the health post in iCCM areas suggested perceptions of a lack of drugs and expectation of poor service at the health post as reasons for not using iCCM services provided by the HEWs—despite a study suggesting significant improvements in these areas.^29^ This suggests that perceptions of quality of services are lagging behind rapid improvements taking place as part of the iCCM scale-up.

There is an urgent need to improve education and demand generation activities in addition to addressing remaining supply-side barriers as part of a more comprehensive iCCM strategy in rural Ethiopia and to target these activities to populations most in need. At present, there are few guidelines for demand generation in the iCCM scale-up strategy.^7^ In Ethiopia, there is a unique opportunity to shift demand generation activities and decrease the workload of HEWs in the Health Development Army initiative—a new cadre of volunteer community health aide consisting of one woman from every five rural households trained in demonstrating “model health behaviors” to their communities.^42^ Furthermore, geographic barriers should be addressed in the siting of health posts, through active case finding and treatment within the households by HEWs and members of the Health Development Army, targeting of distant sub-villages and innovative community-based transportation actions. These activities, especially active case finding and treating children within the home, might potentially reach young children who would not otherwise be taken for treatment outside the household. There is also need for further research on caregiver perceptions of severity, characteristics of the HEW, and service-related factors that might be associated with the use of health post services to get a better understanding of additional determinants of utilization in this setting.

Our research has demonstrated that while the iCCM scale-up is leading to some increases in knowledge and awareness relative to the existing CCM and HEP programs, these factors have not yet led to significant overall improvements in utilization of HEWs delivering iCCM services. It is possible that the effects of Ethiopia's iCCM scale-up have yet to manifest given the relatively short, 2-year, period since the start of the strategy's implementation. There were some promising findings in slightly higher utilization in iCCM areas, some vulnerable groups were more likely to use HEWs delivering evidence-based child health treatments at the health post and caregivers were more likely to go to health posts compared with higher levels of care. On the basis of our findings, it appears that caregiver perceptions about child illness, availability of treatments, and quality of care are critical factors determining utilization of HEWs delivering the iCCM strategy. These factors can and should be corrected through emerging and existing opportunities for demand generation activities—activities often overlooked in iCCM scale-up.^9^ Despite low utilization of appropriate sources of care for sick children, Ethiopia is making rapid and considerable gains in reducing under-five mortality. We would argue that this success could be considerably amplified by focusing on removing existing barriers to utilization and aggressively promoting demand for iCCM services within rural Ethiopian communities.

## Figures and Tables

**Table 1:** Comparison between routine CCM and iCCM guidelines and inputs in Ethiopia

	Routine CCM	iCCM
Management of iCCM illnesses for children 2–59 months		
Pneumonia	Referral to health center	Co-trimoxazole
Severe pneumonia	Referral to health center	Pre-referral treatment with co-trimoxazole
Diarrhea (no/some dehydration)	ORS/ORT	ORS/ORT
		Zinc
Severe diarrhea (severe dehydration, persistent diarrhea, severe persistent diarrhea, dysentery)	ORS	ORS
	Vitamin A (for persistent and severe persistent diarrhea only)	Vitamin A (for persistent and severe persistent diarrhea only)
	Referral to health center	Referral to health center
Malaria	Antimalarial	Antimalarial
Severe febrile disease	Referral to health center	Pre-referral treatment with co-trimoxazole
		Referral to health center
Uncomplicated malnutrition	RUTF or supplementary feeding program	RUTF or supplementary feeding program
Severe complicated malnutrition	Pre-referral treatment with amoxicillin and vitamin A	Pre-referral treatment with amoxicillin and vitamin A
	Referral to health center	Referral to health center
Program inputs		
Training	No additional training	6-day training on iCCM
Supervision	Standard government supervision	Standardized supportive supervision on iCCM supported by partner organizations plus standard government supervision
		Biannual PRCM meetings
Supply of commodities	Standard government commodity supply chain system	Support for purchase and supply of drugs and other commodities by UNICEF and partners
	No additional supplies or job aids	Provision of iCCM registers, iCCM chart booklets, timers, and other supplies
Monitoring and evaluation	Standard government monitoring and evaluation	Enhanced data collection during supervisions and PRCM meetings
		Data management support by UNICEF

iCCM = integrated community case management; ORS/ORT = oral rehydration salts/oral rehydration therapy; PRCM = performance review and clinical mentoring; RUTF = ready-to-use therapeutic food; UNICEF = United Nation Children's Fund.

Source: UNICEF Ethiopia, 2012.

**Table 2:** Selected characteristics of communities, caregivers and their children reported sick with diarrhea, fever, and/or pneumonia in the 2 weeks preceding the survey in Jimma and West Hararghe zones, Oromia Region, Ethiopia, 2013 for total sick children (*N* = 2,248) and by type of child health service available in a caregiver's community

Variables	Total sick children *N* (%)*†	Routine CCM *n* (%)†	iCCM *n* (%)†	*P* value
Number of children	2,248 (100)	1,247 (49.2)	1,001 (50.8)	
Predisposing characteristics				
Religion				0.54
Muslim	1,939 (92.2)	1,053 (91.5)	886 (92.9)	
Christian	224 (7.8)	149 (8.5)	75 (7.1)	
Household size (number of members)				0.30
≤ 3	204 (9.2)	105 (8.3)	99 (10.0)	
4–6	1,133 (48.9)	622 (47.7)	511 (50.0)	
≥ 7	911 (41.9)	520 (44.0)	391 (40.0)	
Maternal age in years				0.50
15–19	98 (4.3)	56 (4.5)	42 (4.2)	
20–29	1,111 (49.9)	617 (49.1)	494 (50.7)	
30–39	769 (36.4)	415 (35.6)	354 (37.2)	
40–49	188 (9.4)	117 (10.8)	71 (7.9)	
Maternal education				0.77
None	1,724 (78.6)	959 (78.2)	765 (79.0)	
Some formal	442 (21.4)	246 (21.8)	196 (21.0)	
Previous use of HP services				0.46
Never used	1,239 (70.2)	695 (71.4)	544 (69.0)	
Used	504 (29.8)	281 (28.6)	223 (31.1)	
Child gender				0.83
Male	1,187 (52.1)	662 (52.4)	525 (51.9)	
Female	1,061 (47.9)	585 (47.6)	476 (48.1)	
Enabling characteristics				
Zone of residence				0.19
Jimma	1,364 (63.7)	747 (61.4)	617 (66.0)	
West Hararghe	884 (36.3)	500 (38.6)	384 (34.1)	
Household distance to the HP in minutes travel time				0.46
0–30	805 (37.7)	471 (39.5)	334 (35.9)	
31–60	591 (26.6)	328 (26.1)	263 (27.0)	
61–120	561 (24.4)	315 (24.8)	246 (23.9)	
> 120	288 (11.4)	131 (9.6)	157 (13.2)	
Household wealth				0.04‡
Poorest 40%	1,083 (44.0)	603 (43.6)	480 (44.4)	
Middle 40%	833 (38.8)	482 (42.0)	351 (35.7)	
Wealthiest 20%	332 (17.2)	162 (14.4)	170 (19.9)	
Awareness of child health services at HP				< 0.01‡
Not aware	1,027 (56.9)	598 (62.7)	429 (51.4)	
Aware	719 (43.1)	381 (37.3)	338 (48.6)	
Need characteristics				
Maternal knowledge of danger signs in number of IMCI signs reported				0.03‡
0	230 (9.4)	151 (11.5)	79 (7.4)	
1–2	908 (39.7)	517 (43.0)	470 (36.6)	
≥ 3	1,110 (50.9)	579 (45.5)	531 (56.1)	
Experienced previous child death				0.93
No	1,441 (66.4)	793 (66.6)	648 (66.3)	
Yes	712 (33.6)	404 (33.5)	308 (33.7)	
Child age in years				0.50
< 1	419 (18.7)	236 (19.9)	183 (17.5)	
1	470 (21.0)	241 (19.4)	229 (22.6)	
2	492 (22.2)	281 (21.9)	211 (22.5)	
3	470 (21.2)	264 (21.4)	206 (21.0)	
4	391 (17.0)	222 (17.5)	169 (16.5)	
Illness type				0.37
Reported diarrhea only	507 (23.2)	280 (23.9)	227 (22.5)	
Reported pneumonia	577 (24.4)	334 (25.8)	243 (23.1)	
Other	1,164 (52.4)	633 (50.3)	531 (52.4)	

iCCM = integrated community case management; HP = health post; IMCI = integrated management of childhood illness.

* The number of missing values may vary for each variable. The percentages presented are valid percentages.

† The percentage is adjusted for sample weight, multistage cluster weight. Therefore, the percentage may not be equal to simple unweighted count.

‡ Statistically significant association in χ^2^ test at *P* < 0.05 level.

**Table 3:** Logistic regression models comparing utilization of the HEW/HP for children reported sick with diarrhea, fever, and/or pneumonia in communities receiving routine CCM and communities receiving iCCM child health services

	Bivariate OR (95% CI)	Multivariate aOR (95% CI)		
		Model 1*	Model 2†	Model 3‡
Routine CCM	1.00	1.00	1.00	1.00
iCCM	1.44 (0.97–2.12)§	1.35 (0.91–2.00)	1.35 (0.91–1.99)	1.33 (0.89–1.97)
	*P* = 0.07	*P* = 0.13	*P* = 0.14	*P* = 0.16

aOR = adjusted odds ratio; CI = confidence interval; HEW = health extension worker; HP = health post; iCCM = integrated community case management; OR = unadjusted odds ratio.

* Model 1 includes predisposing characteristics.

† Model 2 includes predisposing + enabling characteristics.

‡ Model 3 includes predisposing + enabling + need characteristics.

§ Statistically significant association at *P* < 0.10 level.

**Table 4:** Results of bivariate analyses for selected study sample characteristics and utilization of the HEW/HP for children reported sick with diarrhea, fever, and/or pneumonia for the total sample and by type of child health services available in a caregiver's community

Variables	Total	Routine CCM	iCCM
	OR (95% CI)	OR (95% CI)	OR (95% CI)
Predisposing characteristics			
Religion			
Muslim	1.00	1.00	1.00
Christian	1.14 (0.60–2.17)	1.27 (0.62–2.60)	1.06 (0.36–3.10)
Household size (number of members)			
≤ 3	1.00	1.00	1.00
4–6	0.93 (0.50–1.75)	1.81 (0.57–5.78)	0.71 (0.34–1.49)
≥ 7	0.80 (0.42–1.52)	1.61 (0.51–5.09)	0.59 (0.27–1.32)
Maternal education			
None	1.00	1.00	1.00
Some formal	0.79 (0.50–1.26)	0.96 (0.46–2.02)	0.68 (0.38–1.20)
Previous use of HP services			
Never used	1.00	1.00	1.00
Used	6.01 (3.84–9.40)***	5.25 (2.99–9.20)***	6.57 (3.43–12.58)***
Child gender			
Male	1.00	1.00	1.00
Female	1.10 (0.77–1.57)	0.92 (0.55–1.52)	1.24 (0.75–2.05)
Maternal age in years			
15–19	0.53 (0.19–1.48)	0.07 (0.01–0.51)**	0.88 (0.29–2.60)
20–29	1.00	1.00	1.00
30–39	1.02 (0.71–1.47)	1.29 (0.82–2.03)	0.86 (0.49–1.49)
40–49	0.67 (0.32–1.40)	0.98 (0.32–2.60)	0.98 (0.15–1.46)
Enabling characteristics			
Zone of residence			
Jimma	1.00	1.00	1.00
West Hararghe	0.70 (0.47–1.04)*	0.87 (0.51–1.49)	0.60 (0.33–1.07)*
Household distance to the HP in minutes travel time			
0–30	1.00	1.00	1.00
31–60	0.66 (0.42–1.04)*	0.52 (0.42–1.04)*	0.71 (0.40–1.25)
61–120	0.61 (0.39–0.95)**	1.13 (0.62–2.06)	0.33 (0.16–0.68)***
> 120	0.55 (0.30–0.99)**	1.16 (0.55–2.42)	0.28 (0.11–0.71)***
Household wealth			
Poorest 40%	1.00	1.00	1.00
Middle 40%	1.03 (0.71–1.52)	0.87 (0.51–1.50)	1.22 (0.72–2.08)
Wealthiest 20%	1.13 (0.75–1.70)	1.21 (0.58–2.53)	1.05 (0.65–1.71)
Awareness of child health services at HP			
Not aware	1.00	1.00	1.00
Aware	2.52 (1.64–3.84)***	2.31 (1.23–4.35)**	2.52 (1.42–4.46)***
Need characteristics			
Maternal knowledge of danger signs in number of IMCI signs reported			
0	1.00	1.00	1.00
1–2	1.57 (0.72–3.41)	1.31 (0.49–3.52)	2.01 (0.58–6.96)
≥ 3	1.78 (0.86–3.69)	1.17 (0.86–3.15)	2.57 (0.84–7.84)*
Experienced previous child death			
No	1.00	1.00	1.00
Yes	0.79 (0.52–1.19)	0.97 (0.60–1.57)	0.67 (0.35–1.27)
Child age in years			
< 1	0.61 (0.33–1.13)	1.33 (0.59–3.00)	0.29 (0.13–0.66)***
1	1.00	1.00	1.00
2	1.08 (0.62–1.87)	1.55 (0.76–3.15)	0.90 (0.42–1.90)
3	0.85 (0.49–1.47)	1.24 (0.63–2.42)	0.71 (0.33–1.53)
4	0.96 (0.48–1.93)	0.89 (0.37–2.12)	1.07 (0.42–2.72)
Illness type			
Reported diarrhea only	1.00	1.00	1.00
Reported pneumonia	1.30 (0.82–2.05)	0.70 (0.38–1.31)	2.17 (1.12–4.22)**
Other	0.96 (0.60–1.52)	0.68 (0.37–1.25)	1.29 (0.64–2.61)

CI = confidence interval; HEW = health extension worker; HP = health post; iCCM = integrated community case management; OR = unadjusted odds ratio.

* Statistically significant at the *P* < 0.10 level.

** Statistically significant at the *P* < 0.05 level.

*** Statistically significant at the *P* < 0.01 level.

**Table 5:** Results of multivariate logistic regression models predicting likelihood of utilization of the HEW/HP for children reported sick with diarrhea, fever, and/or pneumonia stratified into communities receiving routine CCM child health services and communities receiving iCCM child health services

Variables	Routine CCM available in community			iCCM available in community		
	Model 1† aOR (95% CI)	Model 2‡ aOR (95% CI)	Model 3§ aOR (95% CI)	Model 1† aOR (95% CI)	Model 2‡ aOR (95% CI)	Model 3§ aOR (95% CI)
Predisposing characteristics						
Religion						
Muslim	1.00	1.00	1.00	1.00	1.00	1.00
Christian	1.22 (0.61–2.44)	1.18 (0.58–2.39)	1.23 (0.59–2.57)	1.08 (0.38–3.09)	0.93 (0.35–2.46)	0.82 (0.28–2.45)
Household size (number of members)						
≤ 3	1.00	1.00	1.00	1.00	1.00	1.00
4–6	1.07 (0.31–3.68)	1.10 (0.33–3.67)	1.19 (0.35–4.09)	0.61 (0.28–1.32)	0.62 (0.28–1.38)	0.61 (0.27–1.39)
s≥ 7	0.78 (0.24–2.55)	0.78 (0.24–2.51)	0.84 (0.26–2.73)	0.55 (0.23–1.29)	0.56 (0.23–1.32)	0.56 (0.23–1.35)
Maternal education						
None	1.00	1.00	1.00	1.00	1.00	1.00
Some formal	1.08 (0.50–2.35)	1.03 (0.44–2.43)	1.05 (0.44–2.48)	0.59 (0.34–1.03)*	0.50 (0.29–0.86)**	0.46 (0.26–0.79)***
Child gender						
Male	1.00	1.00	1.00	1.00	1.00	1.00
Female	0.93 (0.56–1.56)	0.92 (0.56–1.53)	0.90 (0.54–1.51)	1.28 (0.76–2.16)	1.32 (0.79–2.22)	1.28 (0.76–2.15)
Maternal age in years						
15–19	0.06 (0.01–0.58)**	0.07 (0.01–0.61)**	0.06 (0.01–0.58)**	0.85 (0.25–2.83)	0.94 (0.29–3.03)	1.23 (0.35–4.29)
20–29	1.00	1.00	1.00	1.00	1.00	1.00
30–39	1.51 (0.84–2.73)	1.46 (0.81–2.65)	1.55 (0.83–2.91)	0.94 (0.49–1.77)	0.93 (0.48–1.80)	0.90 (0.46–1.76)
40–49	1.22 (0.44–3.35)	1.18 (0.43–3.25)	1.32 (0.44–3.91)	0.50 (0.14–1.76)	0.45 (0.12–1.68)	0.46 (0.12–1.71)
Enabling characteristics						
Zone of residence						
Jimma	–	1.00	1.00	–	1.00	1.00
West Hararghe	–	0.87 (0.47–1.61)	0.87 (0.46–1.63)	–	0.58 (0.29–1.18)	0.65 (0.32–1.33)
Household distance to the HP in minutes travel time						
0–30	–	1.00	1.00	–	1.00	1.00
31–60	–	0.51 (0.24–1.05)*	0.51 (0.24–1.07)*	–	0.69 (0.38–1.23)	0.71 (0.41–1.23)
61–120	–	1.05 (0.57–1.93)	1.04 (0.56–1.94)	–	0.31 (0.15–0.63)***	0.31 (0.15–0.69)***
> 120	–	1.14 (0.56–2.34)	1.13 (0.55–2.31)	–	0.29 (0.11–0.75)**	0.29 (0.11–0.73)***
Household wealth						
Poorest 40%	–	1.00	1.00	–	1.00	1.00
Middle 40%	–	0.80 (0.47–1.45)	0.82 (0.46–1.46)	–	0.96 (0.54–1.70)	1.05 (0.57–1.92)
Wealthiest 20%	–	1.16 (0.49–2.76)	1.13 (0.45–2.82)	–	0.87 (0.44–1.71)	1.00 (0.49–2.07)
Need characteristics						
Experienced previous child death						
No	–	–	1.00	–	–	1.00
Yes	–	–	0.83 (0.47–1.47)	–	–	0.74 (0.40–1.38)
Child age in years						
< 1	–	–	1.31 (0.55–3.14)	–	–	0.31 (0.13–0.74)***
1	–	–	1.00	–	–	1.00
2	–	–	1.37 (0.64–2.93)	–	–	0.94 (0.41–2.16)
3	–	–	1.22 (0.59–2.52)	–	–	0.85 (0.39–1.87)
4	–	–	0.84 (0.34–2.07)	–	–	1.26 (0.46–3.50)
Illness type						
Diarrhea only	–	–	1.00	–	–	1.00
Any pneumonia	–	–	0.70 (0.38–1.28)	–	–	2.69 (1.30–5.56)***
Other	–	–	0.68 (0.37–1.26)	–	–	1.52 (0.69–3.32)

aOR = adjusted odds ratio; CI = confidence interval; HEW = health extension worker; HP = health post; iCCM = integrated community case management.

† Model 1 includes predisposing characteristics.

‡ Model 2 includes predisposing + enabling characteristics.

§ Model 3 includes predisposing + enabling + need characteristics.

* Significant at the *P* < 0.10 level.

** Significant at the *P* < 0.05 level.

*** Significant at the *P* < 0.01 level.

**Table 6:** Results of multinomial logistic regression models predicting likelihood of utilization for comparisons between selected sources of care for children reported sick with diarrhea, fever, and/or pneumonia*

Variables	HP/home care only†	HC/home care only	Private/home care only	HC/HP	Private/HP	HC/Private
	aRRR (95% CI)	aRRR (95% CI)	aRRR (95% CI)	aRRR (95% CI)	aRRR (95% CI)	aRRR (95% CI)
Type of child health services						
Routine CCM	1.00	1.00	1.00	1.00	1.00	1.00
iCCM	1.21 (0.81–1.83)	0.79 (0.54–1.15)	0.53 (0.33–0.85)***	0.65 (0.39–1.09)*	0.44 (0.25–0.76)***	1.47 (0.85–2.57)
Maternal education						
None	1.00	1.00	1.00	1.00	1.00	1.00
Some formal	0.84 (0.51–1.39)	1.69 (1.20–2.38)***	2.41 (1.56–3.73)***	2.01 (1.13–3.59)**	2.86 (1.60–5.12)***	0.70 (0.42–1.18)
Child gender						
Male	1.00	1.00	1.00	1.00	1.00	1.00
Female	1.10 (0.76–1.59)	0.80 (0.59–1.08)	0.77 (0.51–1.15)	0.72 (0.47–1.12)	0.70 (0.41–1.19)	1.03 (0.62–1.72)
Zone of residence						
Jimma	1.00	1.00	1.00	1.00	1.00	1.00
West Hararghe	0.66 (0.42–1.05)*	0.75 (0.51–1.10)	1.22 (0.72–2.08)	1.13 (0.66–2.01)	1.84 (0.95–3.57)*	0.61 (0.33–1.14)
Household wealth						
Poorest 40%	1.00	1.00	1.00	1.00	1.00	1.00
Middle 40%	1.00 (0.66–1.51)	1.41 (0.97–2.06)*	1.50 (0.84–2.69)	1.41 (0.84–2.35)	1.50 (0.77–2.92)	0.94 (0.50–1.75)
Wealthiest 20%	0.92 (0.54–1.58)	1.01 (0.56–1.81)	1.03 (0.44–2.39)	1.10 (0.56–2.16)	1.11 (0.43–2.89)	0.98 (0.42–2.30)
Household distance to the HP in minutes travel time						
0–30	1.00	1.00	1.00	1.00	1.00	1.00
31–60	0.54 (0.34–0.87)**	0.54 (0.37–0.80)***	1.19 (0.68–2.08)	0.99 (0.59–1.67)	2.19 (1.11–4.31)**	0.45 (0.24–0.84)**
61–120	0.52 (0.33–0.84)***	0.52 (0.36–0.76)***	1.12 (0.62–2.03)	0.99 (0.56–1.76)	2.14 (1.07–4.28)**	0.47 (0.24–0.91)**
> 120	0.46 (0.24–0.88)**	0.40 (0.19–0.81)**	0.94 (0.41–2.17)	0.87 (0.36–2.09)	2.07 (0.84–5.10)	0.42 (0.15–1.15)*
Experienced previous child death						
No	1.00	1.00	1.00	1.00	1.00	1.00
Yes	0.76 (0.50–1.17)	0.91 (0.66–1.26)	1.09 (0.71–1.68)	1.19 (0.75–1.88)	1.43 (0.80–2.54)	0.83 (0.50–1.40)

aRRR = adjusted relative risk ratio; CI = confidence interval; HC = health center; HEW = health extension worker; HP = health post; iCCM = integrated community case management; OR = unadjusted odds ratio.

† Three multinomial logistic regression models estimated with base categories being alternately 1) home care only, 2) health post, and 3) private.

‡ Home care only also includes no actions taken.

* Significant at the *P* < 0.10 level.

** Significant at the *P* < 0.05 level.

*** Significant at the *P* < 0.01 level.
